# Dendritic Cells and Antiphospholipid Syndrome: An Updated Systematic Review

**DOI:** 10.3390/life11080801

**Published:** 2021-08-09

**Authors:** Kuo-Tung Tang, Hsin-Hua Chen, Tzu-Ting Chen, Nicole R. Bracci, Chi-Chien Lin

**Affiliations:** 1 Division of Allergy, Immunology and Rheumatology, Taichung Veterans General Hospital, Taichung 40705, Taiwan; dirac1982@vghtc.gov.tw (K.-T.T.); shc5555@vghtc.gov.tw (H.-H.C.); 2 Faculty of Medicine, National Yang-Ming Chiao Tung University, Taipei 11221, Taiwan; 3 Ph.D. Program in Translational Medicine and Rong Hsing Research Center for Translational Medicine, National Chung Hsing University, Taichung 40227, Taiwan; 4 Department of Medical Research, Taichung Veterans General Hospital, Taichung 40705, Taiwan; 5 Department of Industrial Engineering and Enterprise Information, Tunghai University, Taichung 40704, Taiwan; 6 Institute of Bioinformatics and Structural Biology and Department of Medical Science, National Tsing Hua University, Hsinchu 30013, Taiwan; s9724072@gm.pu.edu.tw; 7 Department of Biomedical Sciences and Pathobiology, Virginia-Maryland College of Veterinary Medicine, Virginia Polytechnic Institute and State University, Blacksburg, VA 24061, USA; nbracci@vt.edu; 8 Institute of Biomedical Science, The iEGG and Animal Biotechnology Center, National Chung-Hsing University, Taichung 40227, Taiwan; 9 Department of Medical Research, China Medical University Hospital, Taichung 40447, Taiwan; 10 Department of Pharmacology, College of Medicine, Kaohsiung Medical University, Kaohsiung 80708, Taiwan

**Keywords:** antiphospholipid syndrome, autoimmunity, β2-glycoprotein I, dendritic cell, immune tolerance

## Abstract

Antiphospholipid syndrome (APS) is an autoimmune disease characterized by autoreactive B and T cells against β2-glycoprotein I (B2GPI), with vascular thrombosis or obstetrical complications. Dendritic cells (DCs) are crucial in the generation of autoimmunity. Here, we conducted a comprehensive systematic review on the relationship between DC and APS. We performed a literature search of PubMed as of 26 March 2021. A total of 33 articles were extracted. DCs are pivotal in inducing inflammatory responses and orchestrating adaptive immunity. DCs contribute to the local inflammation regarding vascular thrombosis or obstetrical complications. Both B2GPI and antiphospholipid antibodies (aPL) can promote antigen presentation by DCs and the generation or maintenance of autoimmunity. In addition, plasmacytoid DC activation is enhanced by aPL, thereby augmenting the inflammatory response. In line with these findings, DC modulation appears promising as a future treatment for APS. In conclusion, our review indicated the crucial role of DCs in the pathogenesis of APS. Deeper understanding of the complex relationship would help in developing new treatment strategies.

## 1. Introduction

Antiphospholipid syndrome (APS) is an autoimmune disease characterized by the aberrant production of β2-glycoprotein I (B2GPI)-dependent antiphopholipid autoantibodies (aPL, including lupus anticoagulant, anticardiolipin antibodies, anti-B2GPI antibodies, etc.), and it manifests as arterial/venous thrombosis or obstetrical complications [1]. The underlying pathogenesis involves autoreactive T cells and B cells producing these autoantibodies [2]. Vascular APS is associated with detrimental morbidities like stroke, ischemic bowel disease, and even mortality. All these confer a significant disease burden in affected patients [3]. Meanwhile, obstetric APS leads to the morbidity of both the mother and fetus [4]. Unfortunately, treatment for APS is far from satisfactory so far [5]. The treatment paradigm is mainly based on antiplatelet agents and anticoagulants [6], associated with major bleeding risks of 0.57–10% per year [7]. However, 20% of patients with vascular APS develop recurrent thrombosis despite treatment [8]. The treatment strategy also fails in 20–30% of patients with obstetric APS [9]. Immune regulation is another way to manage this devastating disease. Indeed, therapies using hydroxychloroquine, an anti-CD20 monoclonal antibody (rituximab), and an anti-B-cell activating factor (BAFF) monoclonal antibody (belimumab) have shown promising results [10,11,12,13]. To be noted, one of the pharmacological effects of hydroxychloroquine is to increase lysosomal pH and thereby disrupt antigen presentation by dendritic cells (DCs)

DCs are crucial in the elicitation of the adaptive immune response. They pivot the initiation and polarization of T helper responses. It is no surprise that altered DC profiles, like its migration, tissue distribution, phagocytosis, antigen presentation, and cytokines secretion have roles in the generation of autoimmunity [14]. In addition, autoreactive B cells and T cells are of critical pathogenicity in APS, indicating the importance of DCs. DCs have also been implicated in the pathogenesis of vascular thrombosis and obstetric disorders. We have undertaken a comprehensive systematic review on the relationship between DCs and APS, hoping that the new findings on the immunopathogenesis of APS could lead to a novel therapeutic approach.

## 2. Materials and Methods

**Figure 1:**
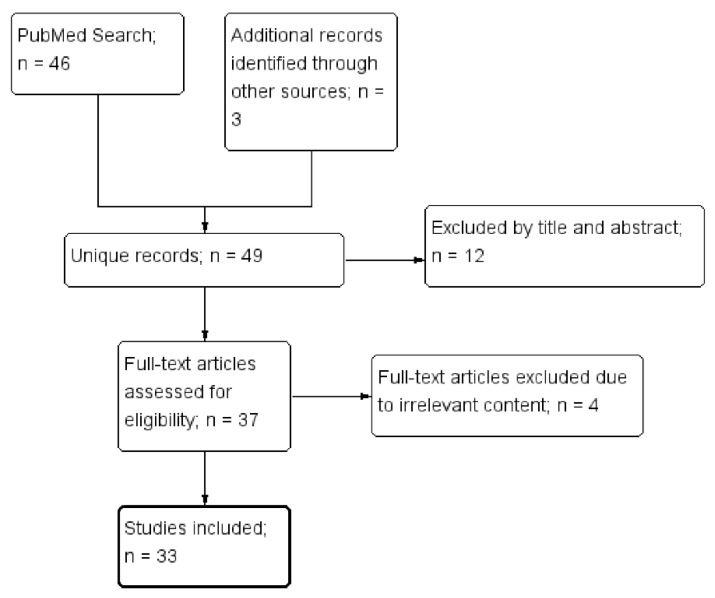
The selection of studies to be included in the systematic review.

This systematic review was on the relationship between DCs and APS. Its review algorithm is shown in Figure 1. We searched MEDLINE on March 26, 2021 using keywords including dendritic cells and antiphospholipid syndrome. The search strategy was as follows: (Dendritic Cells[MeSH] OR Dendritic cell*[tiab] OR Dendritic Cells, Follicular[MeSH] OR Langerhans Cells[MeSH] OR Langerhans*[tiab]) AND (Hughes Syndrome*[tiab] OR Antiphospholipid*[tiab] OR Anti-Phospholipid*[tiab] OR Anti Phospholipid*[tiab]).

Four of the authors (KT Tang, HH Chen, TT Chen, and CC Lin) independently assessed the titles and abstracts as identified by the literature search, retrieving the relevant full-text articles. Two authors (KT Tang and CC Lin) independently assessed the full-text for eligibility of articles and resolved discrepancies through discussion. In addition, the references cited in selected articles were also examined for relevance. Finally, a total of 33 articles were selected.

## 3. Results

### 3.1. Background

#### 3.1.1. The Pathogenesis of APS

The pathogenesis of APS appears elusive, despite some progress in recent decades. In general, two hits are required before disease development [15]. The first hit is the presence of circulating aPL. Cellular and animal experiments showed that anti-B2GPI autoantibodies bound to various receptors, like Toll-like receptors, apolipoprotein endothelial receptor 2, etc., and activated endothelial cells, platelets, and monocytes to sustain a pro-coagulant phenotype in the body, like expressions of tissue factor and thromboxane, etc. [1]. The second hit includes infection or inflammatory events, etc. that can be thrombophilic in triggering the formation of thrombosis in blood vessels. In summary, APS is an immune-mediated thrombotic disorder. Immunomodulation is theoretically a feasible approach for treatment.

#### 3.1.2. Dendritic Cells

DCs are professional antigen-presenting cells located mainly in the peripheral tissues. DCs can be divided into three major subsets: conventional DCs, DCs derived from monocytes, and plasmacytoid DCs [16]. Theses DC subsets induce different types of immune responses [17]. The differences in immunophenotype and function between these DC subsets are shown in Table 1. In general, immature DCs patrol the peripheral tissues to detect pathogens. DCs can capture and process these pathogens, and then they migrate to lymphoid organs, where DCs become mature and present the pathogen-derived antigens to naive T cells to initiate the adaptive immune response [18,19]. Mature DCs are activated through the Toll-like receptor (TLR) signaling pathway, and they produce inflammatory cytokines [20]. Importantly, DCs maturation involves upregulation of costimulatory surface molecules (such as CD80, CD86, CD40, OX40L, and major histocompatibility complex (MHC) class II molecules) and the production of cytokines, chemokines, and proteases [21,22,23]. DCs release distinctive cytokines to activate and regulate T cell differentiation in response to a variety of environmental stimuli. In particular, DCs orchestrate the development of T helper 1 (Th1), Th2, Th17 cells, and regulatory T cell (Treg) responses [24,25]. Several studies have reported critical roles of DC-derived cytokines in the polarization of T helper cells [26]. For example, DCs secrete interleukin (IL)-12 to induce Th1 polarization and IL-4 to induce Th2 polarization from naive CD4+ T cells [27]. The production of IL-6, IL-23, and transforming growth factor (TGF)-β by DCs promotes the differentiation of Th17 cells [28]. Furthermore, DCs secrete IL-10 and TGF-β to induce Treg. These two immunosuppressive cytokines suppress the immune response while promoting a shift to immune tolerance [29]. Thus, DCs have been proposed as key mediators between innate and adaptive immunity. They are important for the induction of either tolerance or immunity against antigens [30,31].

#### 3.1.3. Dendritic Cells and Arterial Thrombosis

There have been a number of studies focusing on the role of DCs in atherosclerosis. Anatomically, DCs are located in the walls of large blood vessels [32]. Both the myeloid and pDC are present in atheroma lesions, typically in rupture-prone areas at the atherosclerotic plaques [33,34]. In patients with acute coronary syndrome or advanced coronary heart disease, circulating myeloid DC precursors reduce in number but increase within the vulnerable plaques [35,36]. Furthermore, in patients with coronary heart disease, the circulating number of pDCs was inversely correlated with the development of cardiovascular events [36]. These findings suggested the recruitment of these cells to the artherosclerotic lesions. Most of these DCs are activated; express CD80, CD86, and inflammatory cytokines; and present in clusters with T cells [33]. Notably, in the blood vessels, immature myeloid DCs interact with activated platelets under low shear stress, a condition resembling that around an advanced atherosclerotic plaque [37]. 

In mice, resident intimal DCs in the artery, similar to macrophages, can take up lipids and contribute to the formation of an atheroma [38]. Moreover, oxidized low-density lipoproteins can stimulate DCs by binding with CD36 and TLR4 to enhance cytokine secretions [39]. A deficiency in costimulatory molecules, like CD80 and CD86, reduces the size of atherosclerotic plaques, supporting the involvement of antigen presentation by DCs [40]. In mice, interferon-β enhances macrophage–endothelial cell adhesion and accelerates atherosclerosis [41]. To be noted, pDC is a rich source of interferon-β. Altogether, accumulating evidence has indicated that DCs may act locally to promote the development of atherosclerosis.

#### 3.1.4. Dendritic Cells and Venous Thrombosis

In this area, the research is scarce. Cherian et al. performed an immunohistochemical examination of veins obtained at operation. They found no DCs in the normal saphenous veins, but they found DCs in veins affected by thrombophlebitis, and the DCs there colocalized with T lymphocytes [42]. Immature myeloid DCs could interact with activated platelets in the areas of blood vessels with low shear stress [37], a condition resembling the venous stasis in Virchow’s triad. These findings imply that DCs promote local inflammation in venous thrombosis, which is similar to the condition in atherosclerosis.

#### 3.1.5. Dendritic Cells and Pregnancy

DCs change their number and function during different stages of pregnancy, which renders it difficult to reach solid conclusions in previous resaerches. Preliminarily, conventional, monocyte-derived, and pDCs have been found in the deciduas in pregnancy [43,44]. Several studies demonstrated an increase in cDCs with or without a decrease in pDCs in blood and deciduas among pregnant women [43,45,46]. Changes in cDCs during pregnancy were accompanied by a predilection toward a Th2 response [43], reduced antigen-specific responses [47], as well as increased Treg cells [46,48,49]. Furthermore, disrupted adaptive changes of cDCs are associated with obstetric disorders, including recurrent spontaneous abortion [46] and preeclampsia [45,50]. Taken together, to sustain gestation, DCs participate in the generation of immune tolerance at the maternal–fetal interface. Its disruption could lead to obstetric complications.

### 3.2. DC in the Pathogenesis of APS

**Figure 2:**
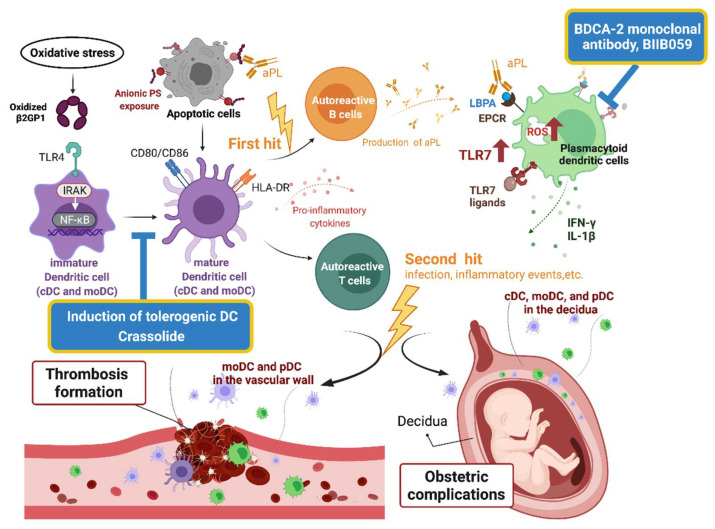
An illustration of the pathogenic role of dendritic cells in antiphospholipid syndrome and potential therapeutics. β2GPI, β2-glycoprotein I; aPL, antiphospholipid antibodies; BDCA, blood dendritic cell antigen; cDC, conventional dendritic cell; EPCR, endothelial protein C receptor; HLA, human leukocyte antigen; IRAK, interleukin receptor-associated kinase; LBPA, lysobisphosphatidic acid; moDC, monocyte-derived dendritic cell; NF-κB, nuclear factor-κB; pDC, plasmacytoid dendritic cell; PS, phospholipids; ROS, reactive oxygen species; TLR, Toll-like receptor.

Accumulating evidence has demonstrated the relationship between DCs and APS. The potential pathogenic role of DCs in APS is summarized in Figure 2. Different DC subsets contribute to the generation and propagation of APS.

#### 3.2.1. B2GPI and Dendritic Cells

B2GPI is a plasma protein that binds to negatively charged phospholipids [51]. In APS, B2GPI is considered a major autoantigen based on the following findings: (a) the detection of aPL requires the presence of B2GPI [52]; (b) immunization of mice with human B2GPI could produce APS-like manifestations, including fetal loss and vascular thrombosis [53]; (c) passive transfer of an anti-BPGPI antibody to mice could produce APS-like manifestations [54]. Interactions between B2GPI and DCs were reported in several studies. Buttari et al found that oxidized B2GPI induced DC maturation, manifested in the expressions of CD80, CD86, and human leukocyte antigen (HLA)-DR and the secretion of inflammatory cytokines [55]. Such interactions involve interleukin receptor-associated kinase (IRAK) phosphorylation and nuclear factor-κB (NF-κB) activation. Furthermore, these DCs display allostimulatory capabilities to prime naïve T cells toward Th1 polarization. A report from Liu et al. is compatible with our recent work, which demonstrated maturation and activation of murine bone marrow-derived dendritic cells (BMDC), isolated from the bone marrow and then cultured with granulocyte–macrophage colony-stimulating factor to induce their differentiation, after B2GPI stimulation [53,56]. These activated DCs then stimulated the proliferation of antigen-specific T cells. Liu et al. further stimulated immature BMDC in TLR4-intact (C3H/HeN) mice with B2GPI. They found greater maturity and higher production of inflammatory cytokines compared with TLR4-defective (C3H/HeJ) mice. Moreover, the ability of BMDC from C3H/HeN mice in stimulating proliferation of allogeneic mixed lymphocytes was higher than that from C3H/HeJ mice. These authors concluded that DC maturation depends on TLR4. Taken together, B2GPI activates DCs through IRAK/NF-κB and TLR4. Higher oxidative stress in APS patients may further promote such activation [57], thereby facilitating the generation and propagation of APS.

#### 3.2.2. Dendritic Cells and Generation of APS

The importance of DCs in the generation of APS has been uncovered in several studies. Apoptosis is implicated in the pathogenesis of APS. Some controversy exists, since exposed anionic phospholipids during apoptosis may provide B2GPI binding sites, which then induce the generation of aPL [58]. Bondanza et al. reported that autoimmunity, including anti-B2GPI IgG, would develop in mice only when apoptotic cells/B2GPI are injected along with syngeneic DCs [59]. Ubiquitin is one of the major pathways for intracellular protein degradation. Kool et al. found that the ubiquitin-editing enzyme, A20, suppresses BMDC activation through the nuclear factor-κB (NF-κB) pathway [60]. A20-deficient DCs enhance the uptake of apoptotic cells and antigen presentation to T cells, leading to the downstream Th1 and Th17 responses. Moreover, these A20-deficient DCs directly stimulate B cells, resulting in their proliferation and differentiation into antibody-producing cells. These authors also genetically deleted A20 in mice and demonstrated in vivo DC activation and expansion and B and T cell activation. In addition, A20-deficient mice develop systemic autoimmunity, including the production of anticardiolipin IgG, thrombocytopenia, and fetal loss, all resembling APS. These findings mimic the generation of APS. Another study by Asano et al. showed that mutated milk fat globule-EGF-factor 8 (MFG-E8), which is originally expressed by DCs and macrophages to bind with phosphatidylserine (an anionic phospholipid) to promote phagocytosis, can inhibit macrophages in the phagocytosis of apoptotic cells [61]. When intravenously injected, the mutant protein induces the production of autoantibodies, including the anticardiolipin antibody, which is enhanced by the simultaneous injection of syngeneic apoptotic thymocytes. However, whether or not APS manifestations developed in these mice were not mentioned. Kuwana et al. initially discovered that CD4+ T cells, autoreactive to a cryptic peptide encompassing amino acid residues 276–290 of B2GPI, are restricted to HLA-DR53+ APS patients. They further treated blood monocyte-derived DCs with B2GPI bound to anionic phospholipids [62]. A co-culture of these DCs and T cells generated autoreactive CD4+ T cells to the same peptide in an HLA-DR-restricted manner ex vivo. Nonetheless, we did not identify any study showing the effect of DC elimination on the generation of APS in mice. A direct link between DC and generation of APS is therefore lacking. Taken together, DC is critically involved in the antigen presentation by apoptotic cells, which could generate autoreactive B and T cells in APS.

#### 3.2.3. Conventional Dendritic Cells and Propagation of APS

Typically, anionic phospholipids exposed on apoptotic cells may exert immunosuppressive signals, and their blockade inhibits the phagocytosis of these cells by macrophages while promoting an inflammatory response [63]. Meanwhile, the blockade of anionic phospholipids has no influence on phagocytosis of these apoptotic cells by DCs, thus shaping a microenvironment facilitating autoantigen presentation and resultant autoimmunity toward cryptic antigens. Antiphospholipid antibodies, through their binding of exposed anionic phospholipids, may theoretically promote this process in the same manner. Furthermore, Rovere et al. reported that aPL could recognize apoptotic cells and bind to their membranes, which opsonized the apoptotic cells to be internalized by DCs [64]. To summarize, the presence of circulating aPL, the so-called “first hit”, could enhance antigen presentation of apoptotic cells by DCs and sustain the autoreactive B and T cells in the body, awaiting the “second hit” to develop vascular thrombosis.

#### 3.2.4. Plasmacytoid Dendritic Cells and APS

The interferon (IFN) signature is the hallmark for several autoimmune diseases, such as systemic lupus erythematosus and Sjogren’s syndrome [65,66]. The signature was detected in 50% of primary APS patients [67] and associated with their endothelial dysfunction [68]. Plasmacytoid DCs are a rich source of type I IFN in the human body. From APS patients, van den Hoogen et al. isolated blood pDCs and showed downregulation of microRNA related to the activated pDCs [69]. In addition, the type I interferon signature was noted in these pDCs, which was correlated with that in blood monocytes. MicroRNA was reduced more markedly in these type I IFN-high pDCs and associated with pDC activation based on the pathway enrichment analyses. Hurst et al. demonstrated that when stimulated with a TLR7 ligand, resiquimod, aPL synergistically induced pDCs to secrete IL-1β [70]. Later, the same research group found that aPL induced the expression of TLR7 and likely its translocation from the endoplasmic reticulum to the endosome in pDC [71]. In line with this, an increased expression of TLR7 was found in peripheral blood mononuclear cells isolated from APS patients. This finding indicated sensitized responses of pDC to TLR7 ligands. In addition, they found that aPL treatment increased RNA uptake in pDC, which further sensitized these cells. Treatment with aPL also induced the generation of reactive oxygen species in the endosomes of pDC. This finding may be related to the activation of DC, since the defective NADPH oxidase, NOX1, could abolish the aPL stimulation of splenic CD11c+ DCs from mice. More recently, they discovered a self-perpetuating cycle, in which aPL signaled through the engagement of the endothelial protein C receptor (EPCR)-lysobisphosphatidic acid (LBPA) on the cell membrane to promote IFN-α production by pDC and downstream expansion of aPL-producing B cells [72]. In summary, pDC could be activated by aPL through multiple mechanisms involving TLR7, reactive oxygen species, and the EPCR–LBPA complex. This may contribute to a pro-inflammatory type I IFN response in APS patients.

### 3.3. Dendritic Cells-Based Therapy for APS

Tolerance induction has emerged recently as a promising treatment strategy for autoimmune diseases [73]. An earlier study on APS mice demonstrated less severe vascular and obstetric complications after being fed with B2GPI. The mechanism was through the induction of tolerance, albeit the role of DC was not clear [74]. Moreover, DCs can be engineered ex vivo using the autoantigen of interest, as well as tolerance-inducing agents, such as 1,25 dihydroxyvitamin D3, dexamethasone, and interleukin (IL)-10 [75]. The resultant tolerogenic DCs were then infused back to humans to induce the Treg response and thereby ameliorating autoimmune diseases. In fact, the therapeutic potential of tolerogenic DC in RA patients has been examined in a human trial, showing some preliminary efficacy with no noticeable side effects [76]. Zandmann-Goddard et al. took a similar strategy in treating APS [77]. They pulsed mouse BMDC with dexamethasone and vitamin D3, as well as B2GPI or its domain I (the major B cell epitope in APS) during lipopolysaccharide-induced maturation. Administration of these tolerogenic DCs suppressed the production of the anti-B2GPI antibody and fetal loss in APS mice, especially with the B2GPI domain I-tolerogenic DCs. Furthermore, adoptive transfer of Treg from these tolerogenic mice could induce tolerance in other APS mice. In addition, Torres-Aguilar et al. produced tolerogenic DCs from blood monocytes isolated from APS patients after treatment with IL-10 and TGF-β [78]. They found that these tolerogenic DCs had induced B2GPI unresponsiveness in autologous effector/memory T cells and with a concomitant increase in Treg cells or greater apoptosis of effector/memory T cells. Could this strategy be utilized for treatment of APS patients? Further human trials are required. To be noted, therapeutic induction of tolerogenic DCs is highly customized, labor-intensive, and costly, which may all limit its clinical application. However, increasingly more medical institutions are equipped with facilities for cell-based therapies. The access of patients to this kind of novel therapy is expected to become more common in the near future. Our recent work utilized a natural compound, crassolide, extracted from soft corals for APS treatment [53]. We found that crassolide had a suppressed activation of murine BMDC ex vivo. The suppression of DCs in APS mice could, in part, translate into their amelioration APS manifestations, including the production of anti-B2GPI antibody, Th1, and Th17 responses toward B2GPI and vascular and obstetric complications. BDCA2 is a pDC-specific receptor, and its binding hinders IFN-response gene expressions. BIIB059, a humanized monoclonal antibody that binds to BDCA2 [79], has been speculated to inhibit the IFN response in patients with autoimmune diseases. Indeed, a pilot study on eight SLE patients demonstrated that a single dose of BIIB059 improved their skin lesions through the suppression of immune cells infiltrate [80]. The results of two phase 2/3 trials in SLE patients are still awaited (NCT02847598 and NCT04895241). Its therapeutic potential for APS should be examined thereafter. Taken together, these findings imply that APS could be effectively treated through DC modulation. However, human trials are needed to prove its efficacy.

## 4. Discussion

DCs are pivotal in the elicitation of an inflammatory response, and they orchestrate the adaptive immunity. DCs contribute to the local inflammation at the atherosclerotic plaque and may also participate locally in the development of venous thrombosis. Moreover, the alterations in DCs may underlie certain obstetric complications, such as recurrent abortion or preeclampsia. In terms of APS, both B2GPI and aPL promote antigen presentation by DCs and generation or maintenance of autoimmunity against B2GPI. In addition, pDCs activation can be enhanced by aPL, thereby augmenting the inflammatory response. In line with the above findings, DC modulation and tolerance induction are promising options in the treatment of APS, as shown in mouse models. Our review has limitations, as most findings are based on mouse models rather than humans. Extrapolating findings to humans should be cautious. In addition, research on the relationship between DCs and APS is relatively scarce amid numerous APS studies, perhaps due to the low number of DCs in mice and humans. Therefore, the pathogenic role of DCs in APS cannot be clearly elucidated based on the literature. Furthermore, no study has investigated the pathogenic role of DCs in APS using either mice with DC elimination or mice lacking functioning DCs. The lack of such direct supporting evidence undermines our conclusions. However, the induction of tolerogenic DCs has indeed been promising for autoimmune diseases in recent years, with the hope of completely reverting the pathogenic process, and producing fewer side effects compared with conventional treatment. It is of great importance to further delineate DCs pathology in APS.

## 5. Conclusions

In conclusion, DCs contribute to both the generation and propagation of APS through their antigen presentation and pro-inflammatory properties. DCs modulation has the therapeutic potential for APS.

## Figures and Tables

**Table 1:** The differences in immunophenotype and function between DC subsets.

DC Subsets	Immunophenotype	Function
Conventional DC1	BDCA-1	Cross-presentation Activation of T helper 1 (Th1), CD8+ T, and natural killer cells
Conventional DC2	BDCA-1, CD11b, CD11c	Cross-presentation Responding to lipopolysaccharide, flagellin, and fungal antigens Activation of Th1, Th2, Th17, and CD8+ T cells
Monocyte-derived DC	BDCA-1, CD11c, CD1a	Contributing to tissue inflammation Activation of Th1, Th17, and CD8+ T cells
Plasmacytoid DC	BDCA-2, BDCA-4, CD123	Responding to viral antigens Production of type I and type III interferons

BDCA, blood dendritic cell antigen; DC, dendritic cell.

## Data Availability

The data that support the findings of this study are available upon request from the corresponding author (C.-C.L.).
